# Genomically personalized therapy in head and neck cancer

**DOI:** 10.1186/s41199-016-0004-y

**Published:** 2016-06-09

**Authors:** Kyaw L. Aung, Lillian L. Siu

**Affiliations:** Drug Development Program, Princess Margaret Cancer Centre, University Health Network, 610 University Avenue, Suite 5-718, Toronto, ON M5G 2M9 Canada

**Keywords:** Cetuximab, PIK3CA Mutation, CCND1 Amplification, PIK3CA Amplification, Tumor Mutation Burden

## Abstract

The current treatment paradigm in head and neck cancer does not adequately address its clinical and biological heterogeneity. Data from genomic profiling studies in head and neck squamous cell carcinoma (HNSCC) have revealed the molecular features that are unique to HNSCC subgroups. This progress in the understanding of HNSCC biology provides an opportunity to develop personalized therapies for patients with distinct molecular subtypes to achieve better clinical outcomes including survival. However there are several well-recognized challenges that need to be overcome before genotype-matched therapies make precision medicine a reality for patients with HNSCC. Selection of appropriate patients for biomarker directed clinical trials based on sound scientific rationale will be critical in making cancer genomics more applicable in this malignancy.

## Background

Head and neck cancer is a heterogeneous disease comprising epithelial tumors of the upper aerodigestive tract. The majority (~90 %) are squamous cell carcinomas arising from the oral cavity, oropharynx, hypopharynx and larynx classed together as head and neck squamous cell carcinomas (HNSCC) [1]. The main risk factors for developing HNSCC are tobacco smoking, alcohol drinking [2] and human papillomavirus (HPV) infection [3]. HPV-positive HNSCC, which is usually found in oropharynx, is a sexually transmitted disease with a rising incidence in many developed countries [4]. It is biologically and clinically distinct from HPV-negative HNSCC that is classically associated with tobacco and alcohol exposure. Patients with HPV-positive disease have a better prognosis compared to those with HPV-negative disease [5].

The standard treatment paradigm for HNSCC is based on anatomical location and stage of the disease. The biological heterogeneity of HNSCC, however, is not routinely incorporated in the current clinical management algorithms. In general, surgery and/or radiotherapy represent the treatment of choice for early stage disease. Surgery is usually preferred for oral cavity tumors, with the need for post-operative adjuvant radiotherapy typically based on recurrence risks that are determined by stage and tumor pathology. Radiotherapy is generally given as primary treatment in oropharyngeal, hypopharyngeal and laryngeal tumors due to the interest in organ preservation, with salvage surgery considered for local recurrences after primary radiotherapy. Concurrent cisplatin-based chemo-radiotherapy is the standard of care for locoregionally advanced disease. For those patients who are deemed inappropriate candidates for platinum-based chemoradiotherapy, radiotherapy combined with the anti-epidermal growth factor receptor (EGFR) monoclonal antibody cetuximab, may be an alternative. The ideal risk-adapted therapeutic strategies aiming to optimize disease control with minimal long-term toxicities in favorable risk early stage disease, and to maximize survival outcomes with acceptable toxicities in poor risk disease, are still evolving.

For recurrent and metastatic HNSCC, platinum-based chemotherapy remains the mainstay of treatment, and addition of cetuximab to first line chemotherapy (cisplatin or carboplatin plus 5-fluorouracil) offers modest survival benefit [6]. Currently, there is no universally agreed second line therapy.

So far, the search for personalized therapy in patients with HNSCC remains elusive. Cetuximab is the only targeted biological agent approved for use in HNSCC and neither *EGFR* copy number nor level of EGFR expression was shown to predict its response [7, 8]. The hope, however, has been that as we sequence broader and deeper into HNSCC genomes, biological drivers in individual HNSCC will be identified with a high precision allowing development of genotype-matching therapy. The emerging data from HNSCC genome sequencing studies [9–13], including recent results from The Cancer Genome Atlas (TCGA) initiative [14], now provide an opportunity to develop genomically personalized therapy for patients with HNSCC.

### Genomic landscape of HNSCC

#### Structural alterations

Genomic structural alterations are commonly seen in HNSCC regardless of HPV status. Both HPV-positive and HPV-negative tumors harbor amplifications of 1q, 3q, 5p and 8q and deletions of 3p, 5q, and 11q [9, 10, 12–14]. The amplification of 3q26/28 region containing squamous lineage transcription factors, *TP63* and *SOX2,* and *PIK3CA* oncogene is seen in both, but more frequently in HPV-positive subtype [9, 10, 12–14]. In HPV-positive tumors, recurrent deletions in *TRAF3* and 11q including *ATM1* and focal amplification of *E2F1* are also seen but 9p21.3 containing *CDKN2A* is usually intact [14]. In contrast, in HPV-negative tumors, 9p21.3 is commonly deleted while 11q13 containing *CCND1*, *FADD* and *CTTN*, and 11q22 containing *BIRC2* and *YAP1* are amplified [14]. It is noteworthy that 7p region that includes *EGFR* is less amplified in HPV-positive tumors [14].

From a biological perspective, recurrent *CDKN2A* deletions and *CCND1* amplification seen in HPV-negative tumors and *E2F1* amplifications in HPV-positive tumors indicate that loss of cell cycle regulation is the fundamental event in HNSCC carcinogenesis. The importance of mitogen activated protein kinase (MAPK) pathway in HPV-negative HNSCC is highlighted by *EGFR* amplification and PI3K-PTEN-AKT-mTOR pathway in both HPV-positive and HPV-negative tumors by *PI3KCA* amplification. Recurrent deletions in *TRAF3* in HPV-positive tumors and amplification of *FADD* and *BIRC2* in HPV-negative tumors showed that NF-kB pathway activation is an important biological driver in HNSCC. *TRAF3* deletions also indicate defective innate immunity response in HPV-positive HNSCC.

### Somatic mutations

#### Genes in cell cycle regulation

Alterations in genes that regulate cell cycle are commonly seen in HNSCC. In HPV-negative tumors, *TP53* is mutated in 80–87 % and *CDKN2A* gene alterations are seen in 32–57 % [11, 12, 14]. *CDKN2A* can also be silenced by promoter hypermethylation in HPV-negative HNSCC [15] and it is noteworthy that CDKN2A expression is lost in almost all HPV-negative HNSCC [16]. On the other hand, *TP53* and *CDKN2A* gene alterations are infrequent in HPV-positive tumors. A small subset of HPV-negative oral cavity squamous cell carcinoma do not have *TP53* mutations but harbor activating *HRAS* mutations and inactivating *CASP8* mutations constituting a distinct subset with a favorable prognosis [14]. *RB1* mutations, although rare at <10 %, are seen predominantly in HPV-positive tumors [11]. *MYC*, in contrast, is amplified in 5–15 % of HPV-negative tumors [11, 14].

#### PIK3CA & PTEN

*PIK3CA* is the most commonly altered oncogene in HNSCC [11, 12, 14]. The presence of hotspot mutations in *PIK3CA* helical domain is a unique feature of HPV-positive tumors, whereas, in HPV-negative tumors, mutations occur throughout the gene despite helical and kinase domain mutations are still common [11, 12, 14]. Twenty one percent of patients in the TCGA cohort had a *PIK3CA* mutation and of those, 25 % also had concurrent *PIK3CA* amplification [14]. An additional 20 % of tumors had *PIK3CA* amplification without mutations [14]. In addition to *PIK3CA* alterations, *PTEN* mutations or deletions are seen in ~11 % of HPV-positive HNSCC and 5 % of HPV-negative HNSCC [11, 17, 18].

#### Genes encoding receptor tyrosine kinase (RTK) and MAPK pathways

HPV-positive and HPV-negative HNSCC have alterations in genes encoding RTK and MAPK pathways at differing frequencies. While alterations in *EGFR, FGFR1,* and *IGF1R* are predominantly seen in HPV-negative tumors, *FGFR2 and FGFR3* alterations including *FGFR3* fusions are more frequent in HPV-positive tumors [12, 14]. *MET* amplification occurs in 2–13 % of HNSCC predominantly in the HPV-negative subtype [19]. *ERBB2* alterations are seen in both subtypes at a low frequency (3–4 %) [14]. Mutations in MAPK pathway, mainly *HRAS* mutations in HPV-negative tumors and *KRAS* mutation in HPV-positive tumors, are seen in ~6 % of HNSCC [12, 14].

#### DNA damage response genes

Mutations in *BRCA1*, *BRCA2* and *ATR* are seen in 6 %, 7 % and 4–10 % of HNSCC respectively [11]. *ATM* mutations are also seen in 1–16 % of HPV-positive HNSCC across studies [11, 12, 14].

#### NOTCH1

*NOTCH1* is one of the most commonly mutated genes in HNSCC (11- 19 %) [9, 10, 14]. Inactivating mutations are predominantly found in HNSCC cases of the TCGA cohort whereas activating gain of function mutations with overexpression of downstream effectors are found predominantly in Chinese HNSCC cases [20–22]. It is well recognized that the biological role of NOTCH could be contextual [23] and NOTCH undoubtedly plays a complex biological role in HNSCC making it challenging to target without further biological insight.

### Patterns of gene expression

In 2002, Belbin et al. first reported that HNSCC could be classified by gene expression patterns (*N* = 17) [24]. Four main subtypes of HNSCC (Group 1–4) based on expression pattern of 12814 genes were subsequently described by Chung et al. (*N* = 60) [25]. These four subtypes were validated independently in the University of North Carolina cohort (*N* = 138) and subtypes 1–4 were named as basal, mesenchymal, atypical and classical respectively [26]. The basal subtype is characterized by activation of EGFR pathway. The mesenchymal subtype has epithelial to mesenchymal transition (EMT) gene expression signature. The atypical subtype mainly consisted of HPV-positive tumors with high expression of *CDKN2A*, *LIG1* and *RPA2*. The classical subtype showed tobacco-induced gene expression signature.

TCGA also independently validated these four signatures (*N* = 278) [14]. Integrated analysis of DNA alterations and RNA expression patterns in TCGA cohort showed that basal subtype tumors had *NOTCH1* inactivation with intact oxidative stress signaling. This subtype also included tumors with *CASP8* and *HRAS* mutations. In contrast, high expression of CD56, a natural killer cell marker, and HLA class I mutations were seen in the mesenchymal subtype. The atypical subtype again consisted mainly of HPV-positive tumors characterized by activating mutations in *PIK3CA* helical domain and a lack of chromosome 7 amplifications indicating a low level of *EGFR* amplification. In contrast, the classical subtype was seen in heavy smokers and at laryngeal site, characterized by *TP53* mutation, *CDKN2A* loss, chromosome 3q amplification and alteration of oxidative stress genes.

These studies, however, suffer from underrepresentation of HPV-positive tumors. Gene expression analysis of a cohort enriched with HPV-positive HNSCC by et al. demonstrated that such tumors could be classified into classical-HPV and mesenchymal-HPV [27]. While both subtypes were characterized by enriched cell cycle genes, classical-HPV also had activation of putrescine (polyamine) degradation pathway possibly related to detoxification of tobacco use and mesenchymal-HPV had enrichment of immune response genes related to the tumoral CD8^+^ T lymphocytes infiltration [27]. HPV-negative mesenchymal tumors in this study also had enrichment of similar immune related genes [27] inferring mesenchymal tumors might respond better to immunotherapy.

### Genomically driven dysregulated pathways and personalized therapies

**Fig. 1:**
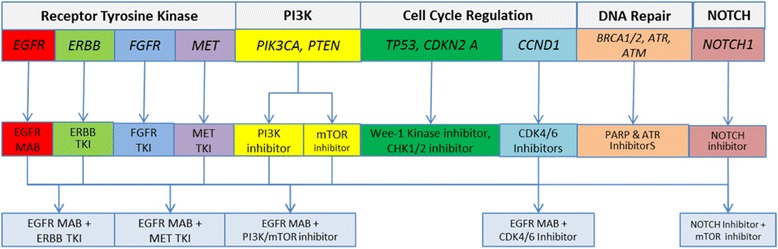
Genomically driven dysregulated pathways and potential matched therapies in HNSCC. Combination strategies currently being tested in ongoing clinical trials are also shown. Abbreviations: MAB, monoclonal antibody; TKI, tyrosine kinase inhibitor

The gene alterations found in HNSCC across studies [9–14] perturb multiple biological pathways. Herein, these pathways are discussed in the context of rationally matched therapies (summarized in Fig. 1).

### TP53 and cell cycle regulation

*TP53* is the most commonly mutated tumor suppressor in HNSCC and almost all HNSCC displayed a dysregulated cell cycle [14]. Loss of cell cycle regulation at G1 to S phase checkpoint is the dominant biological feature in HNSCC. *TP53* and *CDKN2A* loss of function alterations and *CCND1* amplification are the main driving mechanisms of this dysregulation in HPV-negative tumors [11, 12, 14]. In HPV-positive tumors, degradation of TP53 and RB1 by E6 and E7 viral proteins and focal amplification of *E2F1* are the main culprits [12, 14].

To exploit cell cycle dysregulation as a drug target in HNSCC, cyclin dependent kinase (CDK) inhibitors are of particular interest for patients with *CCND1* amplified tumors. CDK4/6 inhibitors, ribociclib (LEE011) and palbociclib, which prolong progression free survival of patients with hormone receptor positive, HER2-negative advanced breast cancer [28, 29], are currently in early phase development in HNSCC (Tables 1 and 2).

**Table 1:** Current active trials in HNSCC with novel single agents^a^

Trial Number	Trial phase	Drug	Pathway targeted	Molecular selection	Setting	Status
NCT02429089	1	LEE011	Cell cycle	No	Recurrent, metastatic	Recruiting
NCT02264678	1, 2	AZD6738	DNA repair	No	Recurrent, metastatic, in combination with chemotherapy or MEDI4736 or olaparib	Recruiting
NCT02567396	1	Talazoparib	DNA repair	No	Recurrent, metastatic	Not yet recruiting
NCT01711541	1, 2	Veliparib	DNA repair	No	Recurrent, metastatic, in combination with chemotherapy	
NCT02365662	1	ABBV-221	EGFR	No	Recurrent, metastatic	Recruiting
NCT01345669	3	Afatinib	ERBB	No	Placebo controlled post-chemoradiotherapy	Recruiting
NCT01415674	2	Afatinib	ERBB	No	Neoadjuvant	Active, not recruiting
NCT01427478	3	Afatinib	ERBB	No	Placebo controlled randomised phase 3, maintenance therapy after post-operative chemoradiotherapy	Recruiting
NCT02131155	3	Afatinib	ERBB	No	Placebo controlled adjuvant trial	Recruiting
NCT02216916	2	HM781-36B	ERBB	No	Recurrent, metastatic	Recruiting
NCT02145312	2	BYL719	PI3K	No	Recurrent, metaststic	Not yet recruiting
NCT02540928	2a	AMG319	PI3K	HPV-negative	Placebo controlled neoadjuvant therapy	Recruiting

^a^Clinical trials with immune checkpoint inhibitors are not included in this Table

**Table 2:** Current active trials in HNSCC testing the safety and efficacy of novel drug combinations

Trials	Trial phase	Drug 1	Drug 2	Pathway 1	Pathway 2	Setting	Status
NCT01716416	1	Pazopanib	Cetuximab	Angiogenesis	EGFR	Recurrent, metastatic	Recruiting
NCT02499120	2	Palbociclib	Cetuximab	Cell cycle	EGFR	Plcebo controlled randomized phase II, recurrent, metastatic	Recruiting
NCT02101034	1,2	Palbociclib	Cetuximab	Cell cycle	EGFR	Recurrent, metastatic	Recruiting
NCT01711541	1, 2	Veliparib	Chemotherapy	DNA repair	EGFR	Recurrent, metastatic	Recruiting
NCT02538627	1	MM-151	MM-121	EGFR	ERBB	Recurrent, metastatic	Recruiting
NCT02501096	1, 2	Pembrolizumab	Lenvatinib	Immune	Angiogenesis	Recurrent, metastatic	Recruiting
NCT02454179	2	Pembrolizumab	ACP-196	Immune	Bruton Tyrosine Kinase	Recurrent, metastatic	Recruiting
NCT02646748	1	Pembrolizumab	INCB039110/INCB050465	Immune	JAK/PI3K	Recurrent, metastatic	Recruiting
NCT01468896	1, 2	Recombinant interleukin-2	Cetuximab	Immune	EGFR	Recurrent, metastatic	Active, not recruiting
NCT02507154	1, 2	NK cells	Cetuximab	Immune	EGFR	Recurrent, metastatic	Recruiting
NCT02643550	1, 2	Monalizumab	Cetuximab	Immune	EGFR	Recurrent, metastatic	Recruiting
NCT02110082	1	Urelumab	Cetuximab	Immune	EGFR	Recurrent, metastatic	Active, not recruiting
NCT02124850	1	Motolimod/Nivolumab	Cetuximab	Immune	EGFR	Stage II-IVA, neoadjuvant	Recruiting
NCT02586987	1	MEDI4736	Selumetinib	Immune	MEK	Recurrent, metastatic	Recruiting
NCT01871311	1	Nilotinib	Cetuximab	Kit	EGFR	Recurrent, metastatic	Recruiting
NCT02277197	1	Ficlatuzumab	Cetuximab	MET	EGFR	Recurrent, metastatic	Recruiting
NCT01332266	1, 2	E7050	Cetuximab	MET	EGFR	Recurrent, metastatic	Recruiting
NCT02205398	1	INC280	Cetuximab	MET	EGFR	Recurrent, metastatic	Recruiting
NCT01285037	1	LY2801653	Cetuximab	MET	EGFR	Recurrent, metastatic	Recruiting
NCT01602315	1b, 2	BYL719	Cetuximab	PI3K	EGFR	Recurrent, metastatic	Active, not recruiting
NCT01488318	2	Dasatinib	Cetuximab	Scr	EGFR	Recurrent, metastatic	Recruiting

In vitro inhibition of G2 to M phase checkpoint enhances apoptosis induced by DNA damaging agents in *TP53* mutant HNSCC [30]. A WEE-1 kinase inhibitor, AZD1775, which disrupts G2 checkpoint by inhibiting CDK1 phosphorylation, is currently being tested in HNSCC in the neo-adjuvant setting in combination with chemotherapy (NCT02508246) and in the locoregionally advanced setting combined with chemoradiotherapy (NCT02585973). Inhibition of checkpoint kinase 1/2 (CHK1/2) also induces mitotic catastrophe followed by cell death in *TP53* mutant HNSCC in vitro [31]. As such combining CHK1/2 inhibitors with radiotherapy or DNA damaging agents seems a rational strategy in HNSCC with *TP53* loss of function mutations. On the other hand, in breast, ovarian, colon and prostate cancer cells, WEE-1 was found to be a synthetic lethal partner of CHK1 and combined inhibition of WEE-1 and CHK-1 results in tumor growth inhibition in vitro and in vivo regardless of *TP53* status [32]. Interestingly, in HPV-positive HNSCC, combining radiotherapy with WEE-1 kinase inhibition increases apoptosis in vitro that is caspase mediated but independent of *TP53* [33]. Currently, the role of *TP53* mutation status in predicting response to WEE-1 and CHK1/2 inhibition remains unclear in HNSCC.

### PI3K-PTEN-AKT-mTOR pathway

*PIK3CA* is the most commonly mutated oncogene in HNSCC and, combined with other gene alterations, PI3K-PTEN-AKT-mTOR pathway is dysregulated in ~30 % of HNSCC [11, 14, 34]. One of the most “actionable” target in this pathway is the activating gain of function *PIK3CA* mutations, as PI3K inhibitors of different classes, either alone or in combination with other targeted agents, are in active development in HNSCC (Tables 1 and 2). On the other hand, loss of function gene alterations in *PTEN* is a challenge to target directly, since the restoration of its tumor suppressive function is not straightforward.

In in vitro studies and patient derived xenografts, *PIK3CA* mutations sensitized response to PI3K and mTOR inhibitors [34, 35]. However, no significant improvement in clinical outcomes was seen with PX-866, an irreversible PI3K inhibitor, in unselected population of HNSCC when combined with doxcetaxel or cetuximab [36, 37]. In particular, 8 patients with *PIK3CA* mutant tumors did not respond to the PX-866 and cetuximab combination raising doubts over the role of *PIK3CA* mutations in predicting response to PI3K pathway inhibition. On the other hand, promising tumor activity was seen with BYL719, a PI3K class I α isoform inhibitor, when combined with cetuximab in a phase Ib study [38] suggesting that efficacy of isoform specific PI3K inhibitors may be different from that of pan-PI3K inhibitors in HNSCC. It is plausible that mutations causing different amino acid substitutions at different positions might have different biological functions with variable sensitivities to PI3K inhibitors [39]. BYL719 is also currently being evaluated with cisplatin-based chemoradiotherapy in locoregionally advanced setting in a phase I study (NCT02537223).

Previous studies demonstrated that mTOR inhibitors, temsirolimus and everolimus, have limited antitumor activity in platinum-refractory recurrent or metastatic HNSCC [40, 41]. Although response rates were higher in platinum naïve setting [42, 43], these studies were performed in non-selected populations and as such the role of *PIK3CA* or *PTEN* alterations in predicting response to mTOR inhibitors in HNSCC is not clearly known.

### Receptor tyrosine kinase pathways

#### EGFR

Overexpression of EGFR was seen in >90 % of HNSCC and associated with a poor prognosis [44–48]. Cetuximab improves survival of patients in both locoregionally advanced setting and recurrent or metastatic setting when combined with radiotherapy and chemotherapy respectively [6, 49]. However, single agent response to cetuximab in recurrent or metastatic HNSCC is 13 % at best indicating there is primary resistance to EGFR inhibition [50].

*EGFR* alterations are found in ~15 % of patients with HNSCC predominantly in HPV-negative tumors [12, 14]. Neither *EGFR* copy number nor level of EGFR expression was shown to predict cetuximab response [7, 8]. *EGFR* mutations including *EGFRvIII* are also extremely rare in HNSCC and unlikely to be useful as predictive biomarkers for EGFR targeted therapy [14, 47, 51–53]. However, alterations in *ERBB2*, *MET*, *PIK3CA, PTEN* and *HRAS* can co-occur with *EGFR* alterations and may explain mechanisms of EGFR inhibitor resistance in individual HNSCC [14]. Combined inhibition of EGFR and other RTK and/or downstream pathways based on genotype of tumors might overcome primary and secondary resistance to EGFR inhibition leading to improved clinical outcomes. Currently, the safety and efficacy of cetuximab combined with multiple molecular targeted therapies are being tested in various phase 1 and 2 clinical trials (Table 2).

#### ERBB

*ERBB2* alteration (amplification plus mutation) is seen in ~4 % of HPV-negative HNSCC and ~3 % of HPV-positive HNSCC [14]. Afatinib, an irreversible pan-ERBB inhibitor, was recently shown to improve progression-free-survival in a non-selected population of recurrent or metastatic HNSCC when compared to methotrexate in the second line setting, indicating that targeting ERBB pathway is a valid therapeutic strategy in HNSCC [54]. Dacomitinib, another irreversible pan-ERBB inhibitor, also demonstrated a single agent response rate of ~13 % in a phase 2 study [55]. Presence of mutations in *PIK3CA* or *PTEN* seems to predict poor PFS in patients treated with dacomitinib in a separate study [56] indicating dual inhibition of ERBB and PI3K pathways might produce better clinical benefit in patients with *PIK3CA* or *PTEN* alterations. A recent phase Ib study has already established maximum tolerated dose of the combination of cetuximab and afitinib [57]. From a genomic perspective, patients with *EGFR* and *ERBB* aberrations are likely to derive better clinical benefit from this novel combination. ERBB targeted agents are currently in active clinical development in HNSCC (Tables 1 and 2).

#### FGFR

*FGFR1* alterations (amplification or mutations) are seen in ~12 % of HPV-negative HNSCC and *FGFR3* alterations (mutations or fusions) in ~11 % of HPV-positive HNSCC [14]. In a preclinical study *FGFR1* mRNA and protein expression level but not *FGFR1* copy number was found to be associated with response to a pan-FGFR inhibitor BGJ398 [58]. Data from squamous cell lung cancer suggest that *FGFR1*-amplified tumor cells with co-expression of MYC are more sensitive to FGFR inhibition [59]. Currently there is no clinical data to indicate the efficacy of FGFR inhibitors in HNSCC as they are still in early stages of drug development.

#### MET

*MET* amplification is found in 2–13 % of HNSCC and it is mutated in ~6 % [19]. Acquired *MET* amplification is a well-recognized biological mechanism of resistance to EGFR inhibition [60–62] and MET overexpression is frequently seen in HNSCC [19]. However, in a phase II study of foretinib, an oral multikinase inhibitor of MET and VEGFR2, in an unselected HNSCC population, no partial or complete response was seen [63]. Despite this disappointing result, combination treatment of MET inhibitors with cetuximab or other RTK inhibitors in selected population is biologically rational and still worth investigating.

### Immune related pathways and genomic predictors for immunotherapy

HNSCC is an immunosuppressive disease and immune checkpoint inhibitors (ICI) are emerging as a promising therapy for patients with HNSCC. Low frequency mutations of *HLA-A*, *HLA-B* and *B2M* are seen in both HPV-positive and HPV-negative tumors implicating that tumor antigen presentation may be disrupted in tumors harboring these genetic alterations [14]. Early data indicate that single agent response to PD-1/PD-L1 pathway inhibition in HNSCC is ~12–20 % [64, 65]. Although objective response rates are higher in patients with PD-L1 positive tumors, PD-L1 expression was not a binary predictive biomarker as PD-L1 negative tumors can demonstrate meaningful tumor shrinkage or clinical benefit [64, 65]. No definite difference in response rate to ICI between HPV-positive and HPV-negative subtypes has been observed [64, 65]. CheckMate 141 (NCT02105636), a phase III study that is comparing the efficacy of nivolumab, an anti-PD-1 inhibitor, in the platinum-resistant recurrent or metastatic setting with investigator’s choice of therapy has been stopped early because it met the primary overall survival endpoint at a preplanned interim analysis [66].

In squamous cell lung cancer, response to nivolumab was shown to associate with tumor mutation burden, neo-antigen load and smoking mutation signature [67]. Although hypermutated phenotype is rare in HNSCC, neo-antigen landscape and smoking signature may be relevant in predicting immunotherapy response in HNSCC. Seventy five percent of HPV-positive HNSCC and 23 % of HPV-negative HNSCC in the combined University of Chicago and TCGA cohort (*N* = 134 + 424 = 558) have an inflamed tumor phenotype [62]. This phenotype is characterized by enrichment of immune response genes related to intra-tumoral CD8^+^ T lymphocyte infiltration, PD-L1 expression and expression of CTLA-4, LAG3, PD-L2 and IDO [68]. There is also a strong correlation between inflamed tumor phenotype and mesenchymal subtype [68]. Considering these emerging data, it is tempting to speculate that HNSCC with this gene expression signature may respond better to ICI. This hypothesis, however, needs further prospective clinical validation.

### DNA damage response and homologous recombination (HR) deficiency

Interestingly somatic mutations in genes involved in DNA damage response including HR are seen in HNSCC at various frequencies [11]. Somatic mutations in *BRCA1* (~6 %), *BRCA2* (~7 %), *ATR* (4–10 %) and *ATM* (1-16 %) are all reported providing the rationale for targeting DNA repair pathway in these tumors [12]. Inactivation of Fanconi anemia/BRCA pathway via promoter hypermethylation of *FANCF* was also seen in 15 % of patients with HNSCC in a previous study [69]. PARP inhibitors and ATR inhibitors, in combination with radiotherapy or cisplatin, are of particular interest for patients with HR deficient genotype. Currently ATR inhibitors, AZD6738 and VX-970, are in early phase of development in HNSCC (NCT02264678; NCT02567422). Furthermore, in a phase I study of AZD1775, a wee-1 kinase inhibitor, a HNSCC patient with a *BRCA* mutation achieved a partial response [70] supporting that DNA repair pathway might be a valid therapeutic target in selected HNSCC population.

### Notch pathway

*NOTCH1* is one of the most commonly mutated genes in HNSCC (~10–15 %) [9, 10, 14] and associated with a poor prognosis [21]. NOTCH pathway plays complex biological roles including cell differentiation, proliferation, angiogenesis and survival [71–73]. As such NOTCH pathway inhibition could be a valid therapeutic strategy in HNSCC. A recent phase I study reported that 2 out of 15 HNSCC patients (13 %) treated with combination of NOTCH inhibitor MK-0752 and mTOR inhibitor ridaforolimus achieved partial response [74]. However, considering the fact that both activating and inactivating NOTCH mutations are seen in HNSCC and the increased risk of squamous cell carcinoma of skin observed with gamma-secretase inhibitors in patients with Alzheimer’s disease [75, 76], targeting NOTCH pathway in HNSCC in the right biological context with minimal risk will be challenging. Further biological insight will be necessary before NOTCH pathway inhibition can be optimally exploited for treatment of patients with HNSCC.

### Challenges & future directions

The recent genomic profiling studies identified biologically distinct HNSCC subgroups providing rationale for developing genomically directed personalized therapies. However, there are well-recognized challenges. Currently available genomic data in HNSCC is limiting as they were mostly derived from early stage resectable and locally advanced tumors with gross underrepresentation of recurrent/metastatic disease (Table 3) and do not truly inform the biological drivers of recurrent and metastatic HNSCC in which most novel targeted agents are currently being tested. Most studies also included only small number of HPV-positive cases (Table 3). Moreover, they were conducted in heterogeneous patient populations without detailed clinical annotation and as such lack power to determine prognostic and predictive value of genetic alterations identified.

**Table 3:** Summary of head and neck cancer clinical samples in genome sequencing studies

Study	Total samples	Oral cavity	Oropharynx	Hypopharynx	Larynx	Other primary sites	Lymph nodes	Metastatic samples	HPV-Positive	HPV-Negative
Stransky et al. [9]	92	51 (55 %)	15 (16 %)	7 (8 %)	15 (16 %)	2 (2 %)	-	-	13 (14 %)	79 (86 %)
Agrawal et al. [10]	32	NK	NK	NK	NK	NK	-	-	4 (12 %)	28 (88 %)
Chung et al. [11]	252	53 (21 %)	12 (5 %)	-	7 (3 %)	103 (41 %)	25 (10 %)	80 (32 %)	84 (33 %)	168 (67 %)
Seiwert et al. [12]	120	23 (19 %)	67 (56 %)	8 (7 %)	19 (16 %)	3 (2 %)	-	-	51 (42 %)	69 (58 %)
Pickering et al. [13]	38	38 (100 %)	-	-	-	-	-	-	NK	NK
TCGA [14]	279	172 (62 %)	33 (12 %)	-	72 (26 %)	-	-	-	36 (13 %)	243 (87 %)

Abbreviation: *NK* not known, *TCGA* The Cancer Genome Atlas

From a biological point of view, the main challenges are those posed by spatial and temporal tumor heterogeneity. Considering profound intra-tumor genetic heterogeneity found in multiple solid tumor types [77–79] including HNSCC [80, 81], it will be difficult to make accurate assessment of tumor genetic characteristics from a single tumor biopsy. To address this in patients with multiple metastatic sites, ideally multiple tumor biopsies from all disease sites will be necessary to assess tumor genomic profile accurately. Cleary, this is not logistically feasible in current oncology practice and innovative tumor sampling methods will be necessary to overcome this challenge. One potential method is mutation profiling of plasma-derived circulating tumor DNA (ctDNA) using next generation sequencing (NGS). As tumor DNA from different metastatic sites are shed into circulation, it could be argued that ctDNA contains mutations or genetic alterations derived from all sub-clones of tumors. With rapid advances in NGS technologies, several groups have demonstrated that molecular characterization of ctDNA is feasible [82–87]. Analysis of serial ctDNA samples could reveal clonal evolution of tumors highlighting the future potential of ctDNA mutation profiling in addressing both spatial and temporal intra-tumor genetic heterogeneity. In appropriate HNSCC cases, it might also be possible to study tumor mutations from DNA isolated from saliva. The feasibility of tumor specific mutation testing from ctDNA and saliva in patients with HNSCC has been explored in a recent study [88].

The obvious drug targets in cancer genomes are activating mutations in driver oncogenes. In HNSCC *PIK3CA* is the most frequently mutated oncogene (~20 %) and PI3K inhibitors are in active development. However, despite the preclinical evidence showing *PIK3CA* mutations sensitize response to PIK3A inhibitors, clinical results so far have been disappointing except possibly for BYL719, a PI3K class I α isoform inhibitor, in combination with cetuximab where some signals of activity have been observed. Further biological insight will be needed to advance development of PI3K inhibitors in HNSCC. Beyond *PIK3CA*, actionable mutations in other oncogenic driver genes such as *ERBB*, *FGFR*, and *MET* are relatively rare making it challenging to conduct biomarker directed clinical trials. Future trials with innovative designs will be needed to address this issue.

Although genetic alterations in tumor suppressor genes *TP53* and *CDKN2A* are common in HNSCC, these alterations are notoriously difficult to exploit for targeted therapeutics at present. Again, mutations in other tumor suppressor genes are seen at low frequencies. Further studies will be needed to elucidate synthetic lethal interactions between these genetic events using computational algorithms such as DAISY (data mining synthetic lethality identification pipeline) [89] or using si-RNA, sh-RNA or CRISPR (clustered regularly interspaced short palindromic repeats) screens so that rational treatment strategies could be developed based on tumor genomic profiles [90]. Currently, one of the most exciting synthetic lethality opportunities involves the use of DNA damaging agents such as PARP inhibitors or ATR inhibitors in patients with HR deficient tumors.

From a genomic perspective, it will be necessary to understand the clonal composition and progression of tumors to develop effective genotype-matched therapy. As clonal selection and progression would have occurred that led to the development of clinically detectable recurrent or metastatic disease, actual clonal composition of advanced HNSCC could be different from that of primary tumors and arguably might contain more genetic driver events that are actionable. Accurate charting of clonal and sub-clonal genetic events in recurrent and metastatic HNSCC based on absolute quantification of somatic mutation events will be critical in finding the relevant therapeutic targets in individual tumors [91, 92]. It is plausible that some genetic alterations are present only in minor sub-clones and targeting those might not produce meaningful clinical benefits.

Considering different biological pathways are active in different subtypes of HNSCC, single agent activity of targeted agents in non-selected population is likely to be modest. To significantly improve clinical outcomes, rational combination treatment strategies should be tested prospectively in selected populations enriched by unique tumor molecular features present in the recurrent or metastatic tumors. Currently, based on available genomic data mainly derived from early stage tumors, the proportion of HNSCC patients who could benefit from personalized therapy remains relatively small considering the most common genetic events in HNSCC occur in tumor suppressor genes. However, concerted large scale genomic profiling programs such as SPECTA (Screening Patients for Efficient Clinical Trial Access) of EORTC (European Organization for Research and Treatment of Cancer) that aims to profile advanced solid tumors to offer genotype-matched therapies could more accurately inform targetable genetic events in recurrent/metastatic HNSCC. These initiatives may also shed insight on the proportion of patients who truly benefit from personalized treatment strategies with acceptable toxicity profiles.

Beyond targeted therapy, ICI are now emerging as a promising new therapy in HNSCC. The genomic predictors of response to ICI clearly exist in other cancer types [67, 93] and it would be prudent to identify genomic and immune mechanisms that underlie tumor immune escape and ICI resistance in HNSCC. As future therapeutic opportunities arise with advances in our understanding of HNSCC biology, there will also be new challenges of translating these advances to personalized therapies. The vision for precision medicine in HNSCC requires concerted interest and continuous effort in the conduct of innovative but complex biomarker directed multidisciplinary trials.

## Conclusion

New treatment paradigms for patients with HNSCC are currently evolving. At present the two main subtypes of HNSCC classified by HPV status remains the most clinically relevant. Development of personalized therapy in HNSCC is still in early stage and results from ongoing preclinical and clinical studies will provide further insight into future novel therapeutic strategies. It is, however, critical that future studies select appropriate patients for potential matched therapies based on sound biological rationale to realize precision medicine in head and neck cancer.

## Abbreviations

CDK, Cyclin Dependent Kinase; CRISPR, Clustered Regularly Interspaced Short Palindromic Repeats; ctDNA, circulating tumor DNA; DAISY, Data Mining Synthetic Lethality Identification Pipeline; EGFR, Epidermal Growth Factor Receptor; EMT, Epithelial Mesenchymal Transition; EORTC, European Organization for Research and Treatment of Cancer; HNSCC, Head and Neck Squamous Cell Carcinoma; HPV, Human Papillomavirus; HR, Homologous Recombination; ICI, Immune Checkpoint Inhibitors; MAPK, Mitogen Activated Protein Kinase; NGS, Next Generation DNA Sequencing; RTK, Receptor Tyrosine Kinase; SPECTA, Screening Patients for Efficient Clinical Trial Access; TCGA, The Cancer Genome Atlas.
